# Potential of «universal» bonding agents for composite repair

**DOI:** 10.1080/26415275.2022.2073234

**Published:** 2022-05-08

**Authors:** Frode Staxrud, Håkon Valen

**Affiliations:** Clinical Research, NIOM: Nordic Institute of Dental Materials, Oslo, Norway

**Keywords:** Composite repair, restoration repair, bonding agent, shear bond strength, composite-to-composite

## Abstract

**Aim:**

The aim of this *in vitro* study was to compare nine different bonding agents of so-called universal type with one well-recognized, 3-step etch-and-rinse bonding agent, as control, in a composite-to-composite shear bond strength (SBS) test.

**Materials and methods:**

Cylindrical composite substrates were made according to manufacturers’ specifications and potted in epoxy according to the description in ISO TS 29022:2003. They were stored in water (37 °C) for 2 months (for water sorption). New composite was bonded to the substrates using nine different bonding agents of universal type, and one 3-step etch-and-rinse bonding agent as control. Fifteen specimens were made for each bonding agent as required by ISO 29022. SBS testing was performed as described in the standard. Vertical load was applied at the speed of 1 mm/min, using a universal testing machine. Two test series were performed: (A) Short term test of SBS after 2 weeks (B) Long term test of SBS after 1-year storage of specimens in water at 37 °C.

**Results:**

Test results for the short term test (A); composite-to-composite SBS mean values ranged from 12.0 to 24.9 MPa, and for the long term test (B), SBS ranged from 11.4 to 25 MPa. Six universal bonding agents showed significantly higher bond strength than control in 1-year test.

**Conclusion:**

In this *in vitro* study, testing shear bond strength of composite-to-composite bonding, the universal bonding agents performed similar and for some agents better than the 3-step etch-and-rinse bonding agent. New simplified bonding procedures seem reliable for repair of composite.

## Introduction

Repair of dental composite restorations is an important part of Minimal Invasive Dentistry [1–4] and satisfying bonding to the old restauration is important for longevity of the repaired restauration. Several new bonding agents have been introduced to the marked during the last decade. As dentists request more simple-to-use-materials, with fewer working steps, potentially minimizing possibilities of operator failure, the manufacturers have responded to this request by simplifying the procedures and combining all ingredients in one bottle, the latest generation so-called ‘Universal bonding agents’.

Almost all manufacturers have launched their own version of a universal bonding agent. They are combinations of one-step bonding systems (primer and bonding in one bottle) and self-etch technologies, based on acidified monomers, mostly 10-MDP (10-methacryloyloxydecyl dihydrogen phosphate). The universal bonding agents may be further subdivided after their acidity in 4 categories (Table 1): UM: Ultra mild (pH > 2.5), M: Mild (pH ≈ 2), IS: Intermediately strong (pH = 1–2) and S: Strong (pH < 1) [5]. It has been questioned whether these all-in-one agents perform as well as the more traditional and well acknowledged multi bottle systems, which have proven their quality for more than 20 years. Earlier, simplified one-step bonding agents have shown to be less efficient over the years than multi bottle systems, when bonding to tooth substance, mainly due to uncertain hydrolytic stability [6]. As reported by Breschi in a review from 2008, a high degree of degradation of the hybrid layer and insufficient resin impregnation of dentin, led to high water permeability of the bonded interface. Sub-optimal polymerization, phase separation, and activation of endogenous collagenolytic enzymes, are factors that may weaken the bonded interface. In contrast, three-step etch-and-rinse and two-step self-etch adhesives continued to show best adhesive results [5,7,8].

**Table 1.** Materials used in the experiments.

Materials used				
Bonding name:	Manufacturer	Batch/LOT	Composition (according to manufacturer)	pH and classification
Optibond FL (control)	Kerr	6605713/Primer:-87/Adhesive:-82	Primer: HEMA, Water, Ethanol Adhesive: GPDM, HEMA, Sodium-hexa-fluoro-silicate. Barium-silicate	Not available
G-Premio Bond	GC	1710132	10-MDP, 4-MET, MDTP, Acetone	1.5 IS
All-Bond Universal	Bisco	180003174	10-MDP, 2-HEMA, BisGMA, Ethanol	3.2 UM
Adhese Universal	Ivoclar	x12305	10-MDP, 2-HEMA, Bis-GMA, other Methacrylates, Ethanol, water, highly dispersed silicon dioxide, initiators, stabilisators	2.8 UM
Optibond Universal	Kerr	6822676	GPDM, HEMA, Acetone, Ethyl Alcohol, Disodium Hexafluorosilicate	2.5 M-UM
Clearfil SE Universal Q	Kuraray	7L0040	10-MDP, Bis-GMA, 2-HEMA, Hydrophilic Amide Monomers, Colloidal Silica, Silane coupling agents, Sodium Fluoride, di-Camphoroquinone, Ethanol, Water	2.3 M
Prime&Bond Elect	Dentsply Sirona	1806000807	10-MDP, Bisacrylamide, Propan-2-ol, Bisacrylamide, Dipentaerythritol pentaacrylate phosphate, 4-(dimethylamino)benzonitrile	2.5 M-UM
One Coat	Coltene	172418	10-MDP, Ethanol, Urethane dimethacrylate, 2-HEMA	2.0–2.8 M-UM
iBond Universal	Kulzer	K010032	10-MDP, Acetone, 4-methacryloxyethyltrimellitic acid anhydride	1.6–1.8 IS
Scotchbond Universal	3M	80513B	10-MDP Monomer, HEMA, Dimethacrylate resins, Vitrebond Copolymer, Filler, Ethanol, Water, Initiators, Silane	2,7 UM
Composite:				
Filtek Supreme XTE	3M	N946833		

The term ‘universal’ refers to different application options. The bonding agents can be used either in ‘etch-and-rinse’ mode (ER) or in ‘self-etch’ mode (SE) [9]. It is also claimed that they can bond to ceramic restorations of glass-type (via silane) and zirconia (via 10-MDP). Nevertheless, there are some shortcomings to be aware of as discussed by van Meerbeek in a recent paper on adhesive technology [5]. Some bonding agents create a very thin bonding film, <10 µm allowing deeper oxygen inhibition, causing poor polymerization through the entire bonding layer, with the consequence that the bonding covering the underlying substrate is not sufficiently polymerized. One can speculate that this poorly converted bonding interface may absorb water from the underlying water-saturated old composite. This may cause hydrolysis of ester bonds. Van Meerbeek suggested that a double layer of bonding agent might partly compensate this problem. Most universal bonding agents contain HEMA (2-Hydroxy-ethyl-methacrylate). HEMA is used for its hydrophilic properties; ability to wet the surface and penetrate moist areas and HEMA can aid prevention of phase separation between hydrophilic and hydrophobic monomers.

Today, there is nearly 10 years of clinical experience with some of these materials (e.g. 3 M Scotchbond Universal). However, little is known regarding their bond strength to composites for repair purposes. Clinical performance is difficult to predict through *in vitro* tests of any kind [10,11], but their relative bond strength should be possible to test with acknowledged laboratory test methods.

### Aim

The aim of this *in vitro* study was to compare nine different new bonding agents of so-called universal type with one well-recognized, 3-step etch-and-rinse bonding agent, often referred to as ‘Gold Standard’ as control, in a composite-to-composite shear bond strength (SBS) test.

The test hypothesis was: there is no difference in shear bond strength between the 3-step etch-and-rinse bonding system and the latest universal bonding agents.

## Methods & materials

### Production of specimens

The materials used and the composition according to the manufacturers are given in Table 1. Test substrates of composite were made by packing composite in plastic rings, using 3 M Filtek Supreme XTE, A3. The substrates were cylindrical; height 3.5 mm, ϴ 8.0 mm and light cured every 2 mm increment, according to manufacturers’ specifications. The light curing unit used was Kerr Demi Ultra with irradiance 1100 mW/cm^2^, as measured by the Norwegian Radiation Protection Authorities, Österaas, Norway. After curing, the substrates were stored in water at 37 °C. One composite (3 M Filtek Supreme XTE) was chosen for substrate, as all the bonding manufacturers claim that their product be compatible to any composite containing bis-GMA (Bowens resin).

All composite substrates were potted in epoxy using cylindrical plastic moulds without covering the surface of the composite according to the description in ISO TS (technical standard) 29022:2013 [12]. After curing of the epoxy, the substrates were stored in water (37 °C) for 2 months (for water sorption), before the composite was ground flat (Fepa # P400) on the side chosen for bonding.

Nine different bonding agents of universal type, and one 3-step etch-and-rinse bonding agent as control (Table 1) were used to bond new cylindrical composite buttons, Ɵ 2.38 mm, to the substrates creating the specimens. The bonding agents were applied according to manufacturers’ instructions for repair. Fifteen, 15, test specimens were made for each bonding agent as required by ISO 29022 (in total 150 substrates). Optibond FL was chosen for comparison (control) because it is a 3-step well recognized bonding agent.

### Shear bond strength testing

Shear bond strength testing was performed according to ISO: 29022:2013 using the equipment described in the standard. The equipment was developed by Ultradent, USA. Modifications were made for composite-to-composite testing as teeth were exchanged with composite substrates. To ensure a clean surface before application of bonding agent, all composite surfaces to be bonded were etched with 37% phosphoric acid, (pH 0.21), rinsed with water, and air dried. The test specimens were fixed vertically in a brass cylinder, and the cylinder was placed in a test jig as described in the standard. Vertical load was applied at the site of the bonding, parallel to the bonded interface, at an overhead speed of 1 mm/min, using a universal testing machine (Instron 1121, Instron Limited, High Wycombe Bucks, UK). The load at fracture, measured in N (Newtons), was converted to MPa (Mega Pascal) calculated from the diameter of the button.

Two test series were performed: (A) Short term test of shear bond strength after 2 weeks storage of specimens in water at 37 °C. Specimens were prepared from substrates in random order. After the test series A was finished, the substrates were ground 0.5 mm at the bonding site, stored in water at 37 °C for 2 months, and randomly reused to produce new specimens for test series B. (B) Long term test of shear bond strength after 1 year storage of specimens in water at 37 °C. A total of 300 specimens were made for test A and B together.

### Statistical analysis

Comparison of proportions of pre-test failures (PTF) was done on the total amount of failures, using the binomial test. Each bonding agent was tested against the control, Optibond FL.

PTFs were excluded from the subsequent bond strength analysis.

Statistical analysis of bond strength results was performed using one-way ANOVA followed by the Dunnet’s *post hoc* test with a significance level of 0,01. Statistical tools were provided by GraphPad Prism 9.3.1. GraphPad Software, San Diego, CA, USA.

## Results

**Figure 1.**
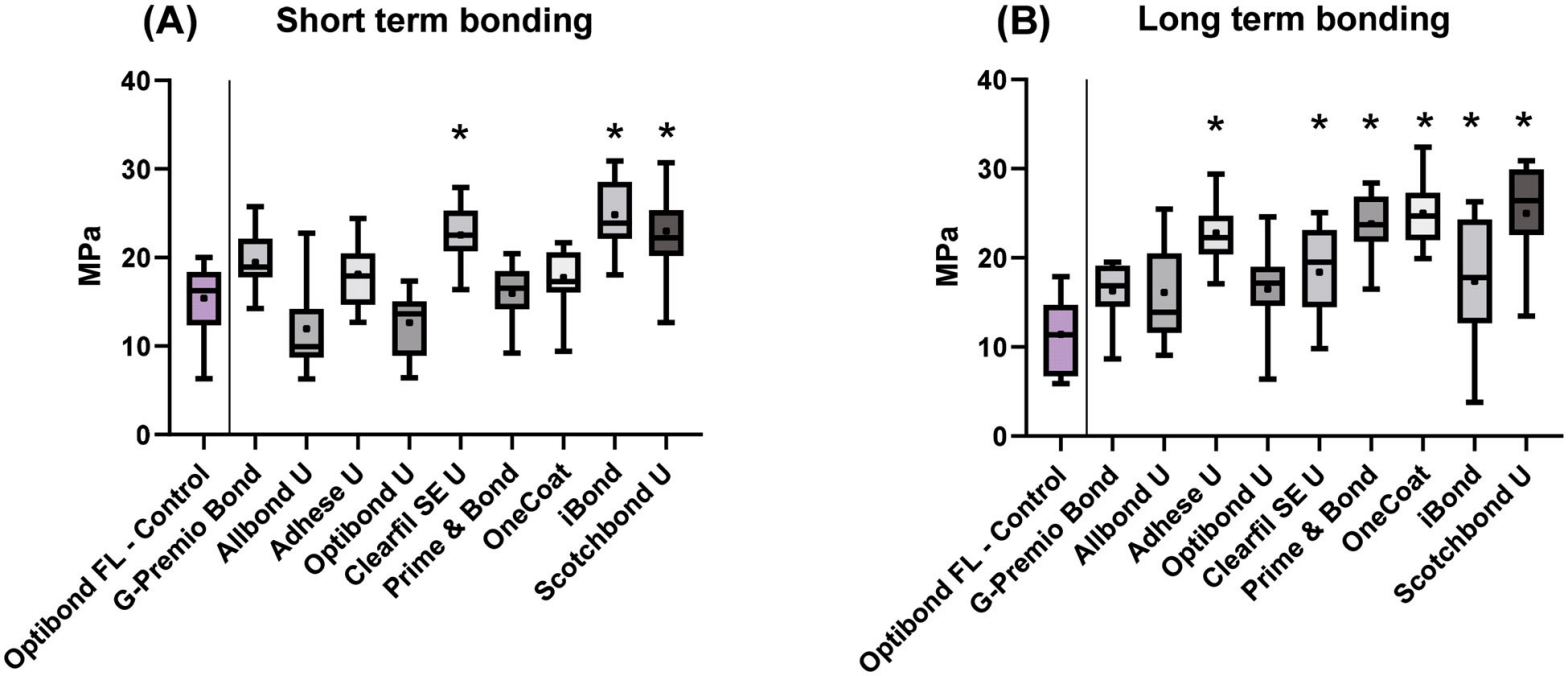
Bond strength of universal bonding agents and control, Optibond FL. (A) 2 weeks storage in water. (B) 1 year storage in water. Box represents the 25 and 75% percentile, solid, horizontal line represents the median, and the dot represents the mean value. Whiskers show minimum and maximum values. Asterisk (*) indicates statistical difference from control, Optibond FL, *p* < .01.

The bond strength results are given in Figure 1. For the short term test (A) the bonding strength range was 12.0–24.9 MPa (mean) and for long term test (B) 11.4–25.0 MPa (mean). After 2 weeks storage, three universal bonding agents showed significantly higher shear bond strength compared to the control, Optibond FL. After 1 year storage in water, six of the universal bonding agents showed significantly higher shear bond strength compared to the control.

The number of PTFs are given in Table 2. The total number of PTFs, i.e. the combined number of failures after 2 weeks and 1 year storage in water, was significantly higher for G-Premio Bond and iBond as compared to Optibond FL. The bonding agents with highest number of PTF seemed to perform well when the bonding agent worked, so the PTF values were consequently excluded in the analyses of the data set.

**Table 2.** Number of pre-test failures (PTF) after 2 weeks and after 1 year (*n* = 15).

Product	2 Weeks (A)	1 Year (B)	Total
Optibond FL (control)	0	3	3
G-Premio bond	4	3	7*
Allbond U	0	2	2
Adhese U	1	0	1
Optibond U	3	0	3
Clearfil SE U	0	0	0
Prime & bond	0	1	1
OneCoat	0	0	0
IBond	5	2	7*
Scotchbond U	0	0	0
Total	13	11	24

Asterisk (*) indicates a significantly higher proportion of PTFs when testing the total amount of failures against the control, Optibond FL.

## Discussion

Are the ‘Universal’ bonding agents something new, or is it the same old stuff in new wrapping? [13]. Dentists search for materials that are simple to use, fast, and reliable. They request fewer working steps which may help reduce handling failures. Universal bonding agents are an attempt to fulfill this demand. Repair of defect restorations is central in the concept of ‘Minimal Invasive Dentistry’ [1,4] and the performance of these bonding agents, in composite-to-composite repair, is of interest. Kanzow performed a test of one universal bonding agent vs. one traditional 3-step etch and rinse bonding agent, both from same manufacturer [14]. They found the universal bonding agent to be more effective than the traditional 3-step bonding agent when testing composite-to-composite and composite-to-amalgam.

The universal bonding agents investigated in the current manuscript, all showed bond strength for composite-to-composite bonding comparable to the ‘Gold Standard’ bonding agent.

The bond strengths observed in the current work in the 1 year test were in the range: 11.4 to 25 MPa (mean), which are higher compared to values obtained in previous tests [15] and in tests of bonding agents to dentine and enamel [13]. All the universal bonding agents in this study showed equal or higher bond strength than the control, Optibond FL (11.4 MPa). This may be of importance as Optibond FL, for decades, has been considered a reliable bonding agent to enamel, dentine and old composite [5].

**Figure 2.**
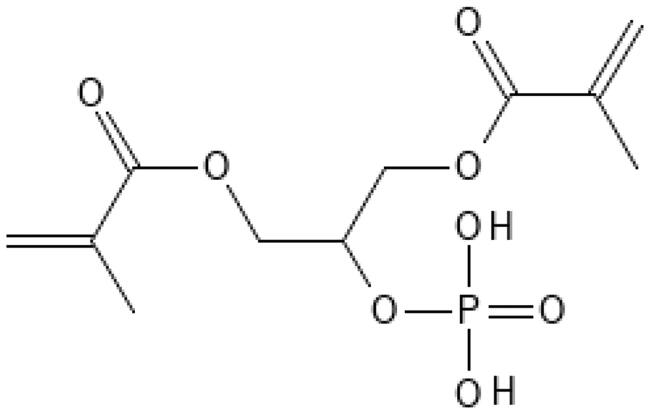
Chemical structure of GPDM.

**Figure 3.**
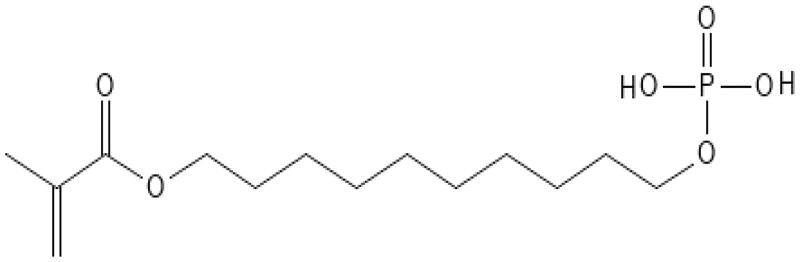
Chemical structure of 10-MDP.

As reported by the manufacturer, the Optibond family were the only bonding agents in this study using Glycerol Phosphate Di-Methacrylate (GPDM) as acidified monomer. All the other bonding agents used 10-MDP (Table 1). Yoshihara showed by XRD (x-ray diffraction) and Nuclear Magnetic Resonance good adsorption of GPDM to hydroxyapatite (HAP) and thereby good bonding properties to tooth structure. However, the water soluble GPDM was easily removed by water spray, unlike MDP which remained adhered to hydroxyapatite (HAP) [16]. It is possible that composite surfaces treated with GPDM become more hydrophilic compared to surfaces treated with MDP. GPDM may absorb more water from the old, water-rich composite, which again could compromise the bonding performance at the composite surface by hydrolysis of ester bonds. The hydrophilicity of GPDM compared to 10-MDP may be explained by their configuration. The former is relatively short and has two hydrophobic methacrylic groups and one hydrophilic phosphate group in the middle (Figure 2). In contrast, 10-MDP has a long hydrophobic spacer chain, separating the functional groups, and making the entire molecule more hydrophobic (Figure 3) [16].

Optibond U, iBond U and G-Premio Bond which all uses acetone as solvent showed lower bond strengths in this study. Van Landuyt found that to avoid phase separation the acetone solvent should be evaporated with a strong air stream [17]. Therefore, it could be that the evaporation of acetone was inadequate, resulting in reduced bond strength.

All solvents should be evaporated entirely to obtain good polymerization of the bonding agents. The polymerization is essential to avoid water uptake from the surface, which subsequently may lead to degradation of ester bonds by hydrolysis.

The use of silanizing agents, used in Scotchbond U, to enhance bonding to ceramic filler particles, seemed to have good effect [18]. A pH >2.5 is needed to stabilize silane in an aqueous solution. Compared to commonly used phosphoric acid (37%, pH 0.21), the high pH of the self-etch universal bonding agents, weakens the etching properties of the bonding solution on enamel. Hence, separate etching of enamel is recommended. When repairing old restorations, it is also recommended to etch the old composite for the purpose of cleaning the surface and to increase surface energy, thus enhancing adhesion properties [19]. To reduce surface contamination, all the composite substrates in the present study were etched with 37% phosphoric acid before application of bonding agent [20].

Application of a separate silane primer has been recommended for bonding to old composites. If used, the silane primer should be freshly mixed and included in the bonding procedure [18,21]. In this paper we were interested in bonding strength of the universal bonding agents per se, and therefore no additional steps were introduced in the protocol. Another reason was that in a survey among Norwegian dentists, approx. 7% of Norwegian dentists reported to use separate silanizing agent when repairing composite restorations [22].

The 10-MDP monomer theoretically form ionic bonds to HAP (Ca^++^). Both ionic- and hydrogen bonds has been reported for bonding to zirconia (used as filler particles in composite). However, the esters in the spacer linking the methacrylate and phosphate functional groups, unfortunately are sensitive to hydrolytic degradation. Concentration and quality (purity) of 10-MDP significantly affect bonding effectiveness [23].

For the analysis on PTF proportions, we could only consider the total number of failures (series A and B combined). As seen in Table 2, Optibond FL had no PTFs in the short term tests (A), and any number of PTFs would by definition be significantly higher. In the long-term tests (B), there were no significant differences between the bonding agents, as the control had the highest number of PFTs. Only when focusing on the total number of PTFs, two universal bonding agents, G-Premio Bond and iBond, had a significantly higher number of PTFs compared to the control. Considering the PTFs, some of the bonding agents might show too favourable bond strength values. However, the reasons for PTFs are unknown, thus for the bonding agents with highest number of PTF, the SBS values should be interpreted with caution.

## Conclusion

In this *in vitro* study, testing shear bond strength of composite-to-composite bonding, the new universal bonding agents performed similar and for some agents better than the ‘Gold Standard’, 3-step etch-and-rinse bonding agent. New simplified bonding procedures seem reliable for repair of composite.

## References

[1] Blum IR, Lynch CD, Wilson NH. Factors influencing repair of dental restorations with resin composite. Clin Cosmet Investig Dent. 2014;6:41–87. 10.2147/CCIDE.S53461 PMC4207439 25378952

[2] Blum IR, Ozcan M. Reparative dentistry: possibilities and limitations. Curr Oral Health Rep. 2018;5(4):264–269. 30524930 10.1007/s40496-018-0191-1 PMC6244566

[3] Ericson D. The concept of minimally invasive dentistry. Dent Update. 2007;34(1):9–10, 12–14, 17–18. 17348554 10.12968/denu.2007.34.1.9

[4] Tyas MJ, Anusavice KJ, Frencken JE, et al. Minimal intervention dentistry-a review. FDI Commission Project 1–97. Int Dent J. 2000;50(1):1–12. 10945174 10.1111/j.1875-595x.2000.tb00540.x

[5] Van Meerbeek B, Yoshihara K, Van Landuyt K, et al. From buonocore's pioneering Acid-Etch technique to Self-Adhering restoratives. A status perspective of rapidly advancing dental adhesive technology. J Adhes Dent. 2020;22(1):7–34. 32030373 10.3290/j.jad.a43994

[6] Peumans M, Kanumilli P, De Munck J, et al. Clinical effectiveness of contemporary adhesives: a systematic review of current clinical trials. Dent Mater. 2005;21(9):864–881. 16009415 10.1016/j.dental.2005.02.003

[7] Breschi L, Mazzoni A, Ruggeri A, et al. Dental adhesion review: aging and stability of the bonded interface. Dent Mater. 2008;24(1):90–101. 17442386 10.1016/j.dental.2007.02.009

[8] Pashley DH, Tay FR, Breschi L, et al. Tezvergil-Mutluay A: state of the art etch-and-rinse adhesives. Dent Mater. 2011;27(1):1–16. 21112620 10.1016/j.dental.2010.10.016 PMC3857593

[9] Rosa WL, Piva E, Silva AF. Bond strength of universal adhesives: a systematic review and meta-analysis. J Dent. 2015;43(7):765–776. 25882585 10.1016/j.jdent.2015.04.003

[10] De Munck J, Mine A, Poitevin A, et al. Meta-analytical review of parameters involved in dentin bonding. J Dent Res. 2012;91(4):351–357. 22173327 10.1177/0022034511431251

[11] Van Meerbeek B, Peumans M, Poitevin A, et al. Relationship between bond-strength tests and clinical outcomes. Dent Mater. 2010;26(2):e100–e121. 20006379 10.1016/j.dental.2009.11.148

[12] ISO: International Organisation of Standardisation. Dentistry – adhesion – notched edge shear bond strength test.; in: ISO/TS 29022:2013 (E). Geneva, ISO/TC 106 Dentistry, SC 1 Filling and restorative materials, 2013.

[13] Chen C, Niu LN, Xie H, et al. Bonding of universal adhesives to dentine-old wine in new bottles? J Dent. 2015;43(5):525–536. 25797702 10.1016/j.jdent.2015.03.004

[14] Kanzow P, Baxter S, Rizk M, et al. Effectiveness of a universal adhesive for repair bonding to composite and amalgam. J Oral Sci. 2019a;61(2):343–350. 31217385 10.2334/josnusd.18-0301

[15] Staxrud F, Dahl JE. Role of bonding agents in the repair of composite resin restorations. Eur J Oral Sci. 2011;119(4):316–322. 21726294 10.1111/j.1600-0722.2011.00833.x

[16] Yoshihara K, Nagaoka N, Hayakawa S, et al. Chemical interaction of glycero-phosphate dimethacrylate (GPDM) with hydroxyapatite and dentin. Dent Mater. 2018;34(7):1072–1081. 29716740 10.1016/j.dental.2018.04.003

[17] Van Landuyt KL, De Munck J, Snauwaert J, et al. Monomer-solvent phase separation in one-step self-etch adhesives. J Dent Res. 2005;84(2):183–188. 15668338 10.1177/154405910508400214

[18] Staxrud F, Dahl JE. Silanising agents promote resin-composite repair. Int Dent J. 2015;65(6):311–315. 26453196 10.1111/idj.12188 PMC9376491

[19] Kanzow P, Wiegand A, Gostemeyer G, et al. Understanding the management and teaching of dental restoration repair: systematic review and meta-analysis of surveys. J Dent. 2018;69:1–21. 28943362 10.1016/j.jdent.2017.09.010

[20] Kanzow P, Wiegand A, Schwendicke F, et al. Same, same, but different? A systematic review of protocols for restoration repair. J Dent. 2019b;86:1–16. 31108118 10.1016/j.jdent.2019.05.021

[21] Yoshihara K, Nagaoka N, Sonoda A, et al. Effectiveness and stability of silane coupling agent incorporated in 'universal' adhesives. Dent Mater. 2016;32(10):1218–1225. 27461880 10.1016/j.dental.2016.07.002

[22] Staxrud F, Tveit AB, Rukke HV, et al. Repair of defective composite restorations. A questionnaire study among dentists in the public dental service in Norway. J Dent. 2016;52:50–54. 27421988 10.1016/j.jdent.2016.07.004

[23] Van Meerbeek B, Yoshihara K, Yoshida Y, et al. State of the art of self-etch adhesives. Dent Mater. 2011;27(1):17–28. 21109301 10.1016/j.dental.2010.10.023

